# Improving Quality-of-Service in Cloud/Fog Computing through Efficient Resource Allocation †

**DOI:** 10.3390/s19061267

**Published:** 2019-03-13

**Authors:** Samson Busuyi Akintoye, Antoine Bagula

**Affiliations:** ISAT Laboratory, Department of Computer Science, University of the Western Cape, Bellville 7535, South Africa; abagula@uwc.ac.za

**Keywords:** CloudSim, virtual machine, greedy heuristics, cloud computing, fog computing, genetic algorithm, data center, CloudSim and hungarian algorithm

## Abstract

Recently, a massive migration of enterprise applications to the cloud has been recorded in the IT world. One of the challenges of cloud computing is Quality-of-Service management, which includes the adoption of appropriate methods for allocating cloud-user applications to virtual resources, and virtual resources to the physical resources. The effective allocation of resources in cloud data centers is also one of the vital optimization problems in cloud computing, particularly when the cloud service infrastructures are built by lightweight computing devices. In this paper, we formulate and present the task allocation and virtual machine placement problems in a single cloud/fog computing environment, and propose a task allocation algorithmic solution and a Genetic Algorithm Based Virtual Machine Placement as solutions for the task allocation and virtual machine placement problem models. Finally, the experiments are carried out and the results show that the proposed solutions improve Quality-of-Service in the cloud/fog computing environment in terms of the allocation cost.

## 1. Introduction

Cloud computing is a computing model to provide on-demand network access to a large pool of networking, storage and computing resources over the internet [1]. This type of computing provides cost reduction because customers do not need to procure hardware for their operations, rather they subscribe for computing resources from the Cloud Service Provider (CSP) only when the cloud services are needed and also only pay for services they consume. Basically, cloud computing is grouped into four deployment models: Private cloud, community cloud, hybrid cloud and public cloud [1,2]. A private cloud is solely owned and managed by an individual organization. In the community cloud model, the cloud infrastructures are owned and shared by various organizations and supports a specific community that has similar operations. The hybrid cloud is a combination of two or more clouds such as public, private and community that remain distinctive entities but are joined together by uniform technology that enables data and applications to be moved easily. In the public cloud model, the cloud infrastructures are made available to the public on a pay-as-you-use basis by the CSP. Broadly speaking, cloud computing is divided into three service models as follows: Platform-as-a-Service (PaaS), Software-as-a-Service (SaaS) and Infrastructure-as-a-Service (IaaS). PaaS allows users to rent virtualized platforms on which to run their own applications or services [3]. In the SaaS model, the cloud customers use the provider’s applications running on a cloud infrastructure. In IaaS, customers are provided with CPU, network bandwidth, storage and other computing resources, all of which they are subsequently able to reconfigure as needed. Invariably, the cloud services are offered to cloud customers and users through the concept of virtualization and distributed computing. Virtualization is an abstraction of computing resources such as storage and processing power to provide virtualized resources called virtual machines (VMs) for customer’s applications [4]. Virtualization technology can be classfied into four groups, namely: Full virtualization, para-virtualization, native virtualization and operating system-level virtualization. Full virtualization provides a full image of the essential hardware. Para-virtualization modifies a guest operating system and is able to communicate directly with the hypervisor [5]. In native virtualization, the guest operating system and host use the same hardware. Operating system-level virtualization does not depend on a hypervisor, rather it modifies the operating system to isolate securely many instances of an operating system within a single host machine. In cloud computing, virtualization technologies such as network, storage and compute offer users an abstraction layer (i.e., VM) that provides a uniform computing platform by concealing the underlying hardware heterogeneity, internal management difficulty and geographic boundaries [4]. When the cloud receives a task request from a user, either a new VM is initialized or an existing VM of the same user is assigned to the request [2]. After the task is processed successfully, every assigned resource is released to the pool of free resources. Sometimes, the number of the available physical resources may not be commensurate with the number of task requests from the cloud user, then resource allocation becomes a critical problem which needs to be solved by CSPs. In such cases, the CSP has the responsibility to determine the number of VMs to be initiated, based on the number of requests from cloud users. The problem of resource allocation in cloud computing involves assigning tasks to VMs and the placement of VMs on Physical Machines (PMs). The computing resource provided is normally allocated based on the Service Level Agreement (SLA) contract between the cloud customers and service providers. The SLA spells out the details regarding the quality of service (QoS) to be offered by the CSP in terms of a range of performance parameters such as reliability, response time, and throughput. The SLA may also specify the payment process and the breach of SLA contract penalty [6]. Thus, the ultimate goal of the CSP is to optimize resource utilization and maximize profit while that of the cloud user is to ensure the cost of leasing the resources is minimized.

### 1.1. Cloud/Fog Computing Resource Management Framework

**Figure 1:**
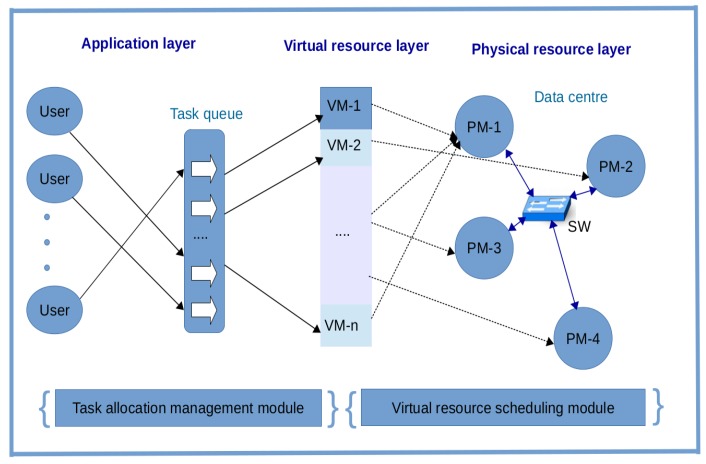
cloud/fog computing resource management framework.

The resource management framework in Figure 1 summarizes the work described in this paper. The multi-layer framework includes:

1. A physical resource layer which is composed of data centres that host PMs in the form of host machines. The PMs are interconnected by switches (SWs).
2. A virtual resource layer which lies above the physical resource layer to virtualize the physical resources as VMs for better resource management.
3. An application layer which lies above the virtual resource layer to provide a variety of services to users. These include SaaS, PaaS and IaaS.

When considered from a service perspective, the framework in Figure 1 can be presented as a two-layer architecture including:

1. A virtual resource scheduling module, where a mapping between physical machines and the virtual machines is made. We assume in this paper that each physical machine (host machine) provides at least one virtual machine.
2. A task allocation management module enabling the virtual resources to be allocated to the users in a cost effective way.

### 1.2. Contributions

This paper proposes an implementation of the framework by assuming that: (i) The tasks and virtual resources are varied and the physical resources are uniform, and (ii) the number of on-demand requests initiated by cloud users is higher than the number of available resources. In this work, we propose a model for assigning the tasks (cloudlets) to VMs, and VM placement with the aim of improving quality of service in the cloud/fog computing environment. The main contributions of this paper are outlined as follows:

- **Problem formulation**: The task allocation and VM placement problem models in the cloud computing environment are formulated and presented. These models aim to minimize the resource allocation cost in a setting where multiple cloud user requests have to be processed on a limited number of physical resources.
- **A task assignment strategy**: We propose the Hungarian Algorithm Based Binding Policy (HABBP) as a heuristic solution to the linear programming problem, and use the algorithm to implement a novel assignment strategy for the famous CloudSim simulator. We also propose the assignment strategy module as a contributed module to CloudSim which includes: (i) A graphical user interface as a front-end component which enables cloud users to interact and communicate with CloudSim and to configure the tasks, VM and PM parameters from the interface, rather than embedding parameter values in the CloudSim source code and (ii) a novel assignment strategy as a back-end component.
- **VMs placement solution**: We propose a Genetic Algorithm Based Virtual Machine Placement (GABVMP) to solve and optimize the VM placement problem in the cloud computing environment.
- **Analysis of experimental results**: We evaluate and compare the performance of the proposed binding policy with the conventional binding policy implemented by the CloudSim simulator and benchmark both solutions against the Simplex algorithm commonly used as a linear programming solver. The proposed GABVMP solution is also compared with the greedy heuristics: Random Placement and First Fit Placement.

### 1.3. Paper Organization

The rest of the paper is arranged as follows: Section 2 presents existing works related to the resource allocation problem in cloud computing. The linear programming model for task allocation is proposed in Section 3. Section 4 presents a task allocation algorithmic solution as a solution for the optimization of the task allocation problem model. Section 5 describes the VM placement problem. In Section 6, the VM placement problem is solved using GABVMP. Section 7 describes the implementation of HABBP and GABVMP, for task allocation and VM placement, respectively. Lastly, we conclude our paper with Section 8.

## 2. Related Work

In cloud/fog computing, resource allocation is the process of assigning available resources to the needed cloud applications over the internet. These resources are allocated based on cloud user request and pay-per-use method. Resources in cloud computing could be either virtual resources or physical resources. Cloud service providers must effectively manage, provide, and allocate these resources to provide services to cloud consumers based on service level agreements (SLAs). Therefore, the appropriate allocation of resources in cloud data centers is also one of the important optimization problems in cloud computing especially when the cloud infrastructure is made of lightweight computing devices.

The quality of service in cloud/fog computing is based on its resource allocation process, and the cloud service provider should assign the resource to the cloud users in an optimal way. The result of any optimal resource allocation strategies must consider certain parameters such as latency, throughput, reduction of energy consumption, minimization of allocation cost and response time. There are many existing works relating to resource allocation in cloud/fog computing. Maguluri et al. [7] propose a stochastic model for resource allocation in cloud computing in which jobs arrive according to a stochastic process and request a variety of virtual machines. The authors use a non-pre-emptive for load balance among the cloud servers and to schedule VM configurations. In order to minimize the communication complexity, the authors consider a distributed system such that each server maintains its own queues. The experimental evaluations reveal that there is only a small difference in delay performance between distributed and centralized queueing systems. Furthermore, the evaluations show that the non-pre-emptive algorithm adopted in this work outperforms the best-fit scheduling algorithm in terms of throughput. Baker et al. [8] present a requirements model for the runtime execution and control of an intention-oriented Cloud-Based Application. The requirements modelling process known as Provision, Assurance and Auditing, and an associated framework are defined and developed where a given system’s functional and non-functional requirements are modelled in terms of intentions and encoded in a standard open mark-up language. An autonomic intention-oriented programming model, using the Neptune language, then handles its deployment and execution. Al-khafajiy et al. [9] propose a fog computing architecture and framework to improve QoS through the request offloading method. The proposed method uses a collaboration strategy among fog nodes in order to permit data processing in a shared mode which satisfies QoS and serves the largest number of IoT requests. The experimental result shows that the performance of fogs layer is significantly increased when the overload is distributed over several fog nodes.

In [10] the author investigates existing resource scheduling algorithms, and classfies them according to some determining factors, such as cost, energy and time. The advantage of the study is that it helps CSPs in the adoption of appropriate scheduling algorithms based on their ultimate goals. Liu et al. [11] propose an earliest finish time duplication algorithm to schedule multiple tasks in heterogeneous data centres. The algorithm can also be referred to as a directed acyclic graph based scheduling algorithm. The performance evaluation of the study reveals that the combination of pre-processing the cloud resources before scheduling and the proposed algorithm, performs better than the heterogeneous earliest finish time algorithms, in terms of task scheduling time. In [12] the authors propose a virtual cloud resource allocation model based on constraint programming to improve the Quality-of-Service (QoS) in cloud computing and decrease the cost of resource utilization. Moreover, the authors [13] propose a VM Repacking Scheduling Problem (VRSP) to minimise the energy consumption while placing VM in the data centres. The benefit of the study is that it is flexible, it enables users to generate automatically the SLA constraints, and it reduces energy utilization.

In order to address the VM placement problem in a data centre, the authors [14] propose a greedy-based algorithm to reduce resource usage, the network traffic and the number of cloud servers. The work divides traffic flows and routes them through two link-disjoint paths to decrease congestion, at the same time meeting the requirements for protection grade as well as bandwidth. Furthermore, the authors [15] propose an online heuristic-based VM placement algorithm which is based on a multi-dimensional space partition model. The objective of the work is to make a trade-off between balancing multi-dimensional resource usage and reducing the number of the PMs used for VM placement. The advantage of the algorithm is that it reduces the number of running PMs as well as the total energy consumption. In [16], authors propose an ant-colony based optimization model with the aim to optimize resource utilization and total power consumption concurrently. The model performs better than the previous multi-objective VM placement algorithm. Pascual et al. [17] propose multi-objective evolutionary algorithms to solve the placement problem. The objectives of work are: (i) The consolidation of VMs on a small set of processors, and (ii) the minimization of associated energy costs for servers and network equipment. The algorithms were implemented using a Flat Tree topology and tiered applications, such as a web server with an associated database. The major advantage of the algorithms is that they enhance the application performance and energy consumption. The work in [18] proposes algorithms for the placement of precedence-constrained parallel virtual machines. The aim of the work is to reduce energy consumption by consolidating virtual machines on the available physical machines yet not degrading the makespan. The algorithms were evaluated using benchmarks of real-world distributed applications and they achieved efficient results.

Georgiou et al. [19] propose VM placement algorithms for the Portland network architecture with the aim to allocate communicating virtual machines in physical proximity to avoid the creation of network bottlenecks. The authors propose two algorithms: the first algorithm is proposed for rapid placement of closely located virtual machines, while the second algorithm is designed to identify network regions that can best host the virtual machines and then, using the first algorithm, maps these virtual machines on the servers. The benefit of the approach is that it has the capability to reduce the intensity of traffic in the links of top-level switches.

Meng et al. [20] propose a Cluster-and-Cut algorithm to improve the scalability of data center networks with traffic-aware VM placement. The goal of the algorithm is to reduce network traffic among VMs and related communication cost by placing inter-communicating VMs in the same PM. The VM placement problem is formulated as a quadratic assignment problem (QAP) to find a suboptimal placement which minimizes network traffic, considering the associated communication cost and a static-single path routing. The allocation cost is defined as the number of switches between two inter-communicating VMs and each PM is divided by slots with the capacity to accommodate a single VM with the assumption of an equal number of VMs and slots. If the number of VMs is lower than the number of slots, dummy VMs are introduced with zero traffic which has no significant effect on the solution of the problem. The performance evaluations of the algorithm show a significant performance improvement compared to existing genetic algorithmic methods.

Breitgand et al. [21] investigate the problem of placing images and VM instances on the servers with the aim to increase the affinity between them to mitigate communication overhead and latency. The problem is modelled as an extension of the Class Constrained Multiple Knapsack problems (CCMK) and present a polynomial time local search algorithm for the same size images. Specifically, this model focuses on an off-line placement problem, where there are a given set of demands and available servers. In order to solve this problem, the local search algorithm was applied as a basis for ongoing optimization which periodically improves the VM placement and greedy placement of a new set of VM instances by allowing migrations of the VMs.

Vakilinia et al. [22] propose a platform for virtual machine (VM) placement/migration to minimize the total power consumption of cloud data centers (DCs). The platform is divided into two parts. Firstly, an estimation module is introduced to predict the incoming load of the DC. Secondly, two schedulers are designed to determine the optimal assignment of VMs to the PMs. The proposed schedulers apply a column generation method to solve the large-scale optimization problem in conjunction with the cut-and-solve-based algorithm and the call back method to decrease the complexity and the time to obtain the optimal solution. The trade-off between optimality and time is investigated. The numerical results show that the proposed platform produces the optimal solution for a limited time-frame. Selmy et al. [23] present virtual machines migration and selection policies to reduce the power consumption of servers in the cloud computing environment. The authors propose neural networks for classification and prediction, Self Organizing Map (SOM) and K-Means Clustering algorithms for the policies. The results of implementation of the proposed policies show significant reduction of energy consumption of the servers in the data center.

All the works mentioned above have been able to solve one or two problems of VM placement in the cloud computing environment. There is still much to be done, however, to mitigate the effect of these problems.

## 3. Task Allocation Problem Model

In this section, we present a linear programming problem model for assigning task requests (cloudlets) from cloud users to VMs. To express the model mathematically, we consider a set of VMs represented by $vm_{i}$ for i≤n and n>1. Similarly, a set of tasks (cloudlets) corresponding with each on-demand user request (job) represented by $\tau_{j}$ for j≤m and m>1. In this work, we assume a one-to-one allocation model where each VM executes only one cloudlet and each cloudlet needs to be assigned to only one VM. However, a many-to-one allocation model may also be considered where several tasks are allocated to a single virtual machine. The many-to-one allocation model is not considered in this paper.

Furthermore, we set n=m and $C=[\omega_{ij}]$ to be an n×n matrix in which $\omega_{ij}$ is the cost of assigning $vm_{i}$ to $cloudlet_{j}$, i.e.,

(1) $$\omega_{ij}=\frac{f_{j}}{vm_{i}}.$$

We also set $\chi =[\alpha_{ij}]$ to be the n×n matrix where

(2) $$\alpha_{ij}=\left\{\begin{array}{cc} 1, & \mathrm{if}\;vm_{i}\;\mathrm{is}\;\mathrm{assigned}\;\mathrm{to}\;cloudlet_{j},\\ 0, & \mathrm{if}\;vm_{i}\;\mathrm{is}\;\mathrm{not}\;\mathrm{assigned}\;\mathrm{to}\;cloudlet_{j}. \end{array}\right.$$

Our goal is to minimize the total cost ρ(χ), defined as the sum of the cost of assigning cloudlets to the available VMs. Thus, we present an optimization problem as a linear programming model in terms of a function ρ as follows:

(3) $$\mathrm{minimize}\;\rho \left(\chi \right)=\sum_{j=1}^{m}\sum_{i=1}^{n}\omega_{ij}\alpha_{ij}$$

subject to the following constraints

(4) $$\begin{array}{ccc} \sum_{i=1}^{n}\alpha_{ij} & = & 1,\;\mathrm{for}\;j=1,2,\ldots ,m, \end{array}$$

and

(5) $$\begin{array}{ccc} \sum_{j=1}^{m}\alpha_{ij} & = & 1,\;\mathrm{for}\;i=1,2,\ldots ,n, \end{array}$$

such that

(6) $$\begin{array}{ccc} \alpha_{ij} & = & 0\mathrm{or}1. \end{array}$$

Thus, any matrix satisfying the Equations (4) and (5) is a solution and conforms to a permutation σ of a set N={1,2,…,n} generated by setting σ(i)=j if and only if $\alpha_{ij}=1$. In addition, if χ is a solution relating to σ, then

(7) $$\sum_{j=1}^{n}\omega_{ij}\alpha_{ij}=\omega_{i\sigma (i)}.$$

summation over *i* from 1 to *n*, we obtain

(8) $$\sum_{i=1}^{n}\omega_{i\sigma (i)}=\sum_{i=1}^{n}\sum_{j=1}^{n}\omega_{ij}\alpha_{ij}.$$

Hence, any solution χ on which ρ(χ) is minimal is known as an optimal solution. We can reform a given allocation problem specified by *C* into another one specified by a matrix $\bar{C}=\left[{\bar{\omega }}_{ij}\right]$, in which ${\bar{\omega }}_{ij}\geq 0$, ∀ pairs i,j, where the two problems have the equal set of optimal solutions. If $\chi^{*}$ is an optimal solution to the problem given by $\bar{C}$, then it is important to know that $\chi^{*}$ is also an optimal solution to the one given by C.

Theorem 1 illustrates the steps to reform a matrix into another with the same set of optimal solutions.

**Theorem** **1.** *A solution X is an optimal solution for $p\left(X\right)=\sum_{i=1}^{n}\sum_{j=1}^{n}c_{ij}x_{ij}$ if and only if it is an optimal solution for $\bar{p}\left(X\right)=\sum_{i=1}^{n}\sum_{j=1}^{n}{\bar{c}}_{ij}x_{ij}$ where ${\bar{c}}_{ij}=c_{ij}-u_{i}-v_{j}$ for any of $u_{1},\ldots ,u_{n}$ and $v_{1},\ldots ,v_{n}$ and $u_{i}$ and $v_{j}$ are real numbers for all i and j.*

**Proof.**  We establish that the difference between the functions p(X) and $\bar{p}\left(X\right)$ is constant $\sum_{i=1}^{n}u_{i}+\sum_{j=1}^{n}v_{j}$.

$$\begin{array}{c} \left.\begin{array}{cc} \bar{p}\left(X\right) & =\sum_{i=1}^{n}\sum_{j=1}^{n}{\bar{c}}_{ij}x_{ij},\\ & =\sum_{i=1}^{n}\sum_{j=1}^{n}\left(c_{ij}-u_{i}-v_{j}\right)x_{ij},\\ & =\sum_{i=1}^{n}\sum_{j=1}^{n}c_{ij}x_{ij}-\sum_{i=1}^{n}\sum_{j=1}^{n}u_{i}x_{ij}-\sum_{i=1}^{n}\sum_{j=1}^{n}v_{j}x_{ij},\\ & =\sum_{i=1}^{n}\sum_{j=1}^{n}c_{ij}x_{ij}-\sum_{i=1}^{n}\sum_{j=1}^{n}u_{i}x_{ij}-\sum_{j=1}^{n}\sum_{i=1}^{n}v_{j}x_{ij},\\ & =p\left(X\right)-\sum_{i=1}^{n}u_{i}\sum_{j=1}^{n}x_{ij}-\sum_{j=1}^{n}v_{j}\sum_{i=1}^{n}x_{ij}. \end{array}\right. \end{array}$$

From Equations (4) and (5),

$$\begin{array}{c} =p\left(X\right)-\sum_{i=1}^{n}u_{i}-\sum_{j=1}^{n}v_{j}. \end{array}$$

This shows that, $p\left(X\right)-\bar{p}\left(X\right)=\sum_{i=1}^{n}u_{i}+\sum_{j=1}^{n}v_{j}.$ Therefore, a solution *X* minimizes p(X) if and only if it minimizes $\bar{p}\left(X\right)$. ☐

## 4. Task Allocation Algorithmic Solution

In this section, we present a task allocation algorithmic solution that is based on Hungarian algorithm [24,25] known as Hungarian Algorithm Based Binding Policy (HABBP) to solve the task allocation problem in the cloud computing environment.

### 4.1. Notation and Preliminaries

- Given a cost-matrix ωm of size n×m,
- *n* is the number of VMs,
- *m* is the number of cloudlets,
- $\omega m_{ij}$ represents the time required to complete $cloudlet_{i}$ by $vm_{j}$.

### 4.2. Procedures of the Algorithm

Algorithm 1 represents the pseudo-code of the HABBP for tasks-to-VMs allocation in a cloud computing environment. The first four lines (1–4) in the algorithm represent the initialization of different variables. Subsequently, we initialize *cost-matrix* by dividing the cloudlet length by the MIPS of VM. In the case of many-to-one allocations where the number of cloudlets and the number of VMs are not equal, we then add the dummy cloudlets/VMs to turn the *cost-matrix* into a square matrix.

**Algorithm 1:** Computation of the total assignment cost C.

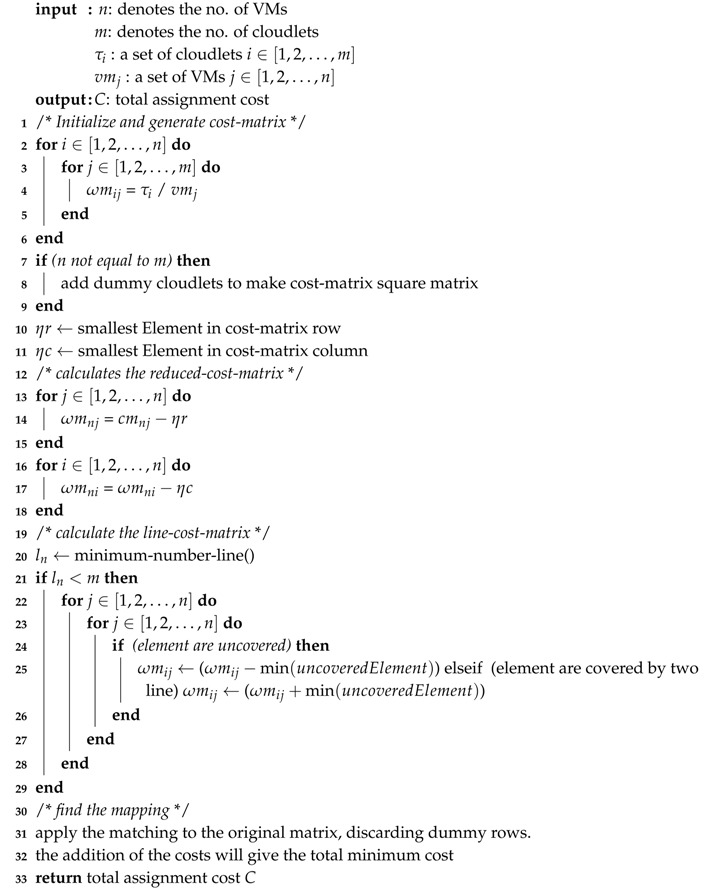



Thereafter, we compute the *reduced-cost-matrix* from the *cost-matrix* by subtracting the minimum value of each row from the elements of its row, turning each minimum value into zero, and by subtracting the minimum value from the elements of each column, turning the minima into zeros. From that, we compute the *line-cost-matrix*. If the number of lines is not equal to the number of VMs, we then subtract the minimum uncovered element from every covered element. If an element is covered twice, we then add the minimum element to it.

Lastly, we apply the mapping to the original matrix, discarding dummy rows, and we add the cost of assigning cloudlets to VMs to give the total minimum cost *C*.

### 4.3. Illustration

We illustrate, through an example, the concept of the HABBP and the steps that need to be followed in order to optimize the assignment of tasks to virtual resources in the cloud computing environment. Table 1 depicts three cloudlets in the queue with a broker and Table 2 depicts VMs initiated in the data centre.

*The algorithm works as follows:* We initialize the *cost-matrix* by dividing the *length* of the cloudlet by the *mips* of the VM as depicted in Table 3.

In this case, the number of cloudlets is equal to the number of VMs. Thus, we do not need to add the dummy cloudlet/VM values to turn the *cost-matrix* into a square matrix. We compute the *reduced-cost-matrix* by subtracting the minimum value of each row and column from the row and column of the *cost-matrix* to yield Table 4 and Table 5 respectively.

Then, we calculate the *line-cost-matrix*; this represents the lines that cover all zeros in the *reduced-cost-matrix*. In this case, there are two lines. The lines are on column 1 and row 2 of the *reduced-cost-matrix*. Since the number of lines is not the same as the number of VMs, we remove the lowest of all uncovered elements from all uncovered elements as indicated in Table 6.

Again, we calculate the minimum number of lines required to cover all zeros in the matrix. The lines are on column 1, row 2 and row 3 of the *reduced-cost-matrix*. Since the number of lines is the same as the number of VMs, an optimal assignment exists among the zeros in the *reduced-cost-matrix*. Therefore, $cloudlet_{1}$ is assigned to $vm_{1}$, $cloudlet_{3}$ is assigned to $vm_{2}$, and $cloudlet_{2}$ is allocated to $vm_{3}$ as indicated in Table 7.

The total cost of assigning cloudlets to virtual machines optimally is: 100 s + 120 s + 160 s = 380 s.

Alternatively, we solve the example mentioned above using Simplex method [26,27] and compare the result with the one that is already generated using HABBP. Using the Equations (3)–(6), the optimization objective function can be formulated as:

(9) $$\mathrm{minimize}\;\rho \left(\chi \right)=100\alpha_{11}+200\alpha_{12}+300\alpha_{13}+40\alpha_{21}+80\alpha_{22}+120\alpha_{23}+80\alpha_{31}+160\alpha_{32}+40\alpha_{33}$$

subject to the following constraints

(10) $$\begin{array}{c} \alpha_{11}+\alpha_{12}+\alpha_{13}=1,\\ \alpha_{21}+\alpha_{22}+\alpha_{23}=1,\\ \alpha_{31}+\alpha_{32}+\alpha_{33}=1,\\ \alpha_{11}+\alpha_{21}+\alpha_{31}=1,\\ \alpha_{12}+\alpha_{22}+\alpha_{32}=1,\\ \alpha_{13}+\alpha_{23}+\alpha_{33}=1 \end{array}$$

and

(11) $$\alpha_{11},\alpha_{12},\alpha_{13},\alpha_{21},\alpha_{22},\alpha_{23},\alpha_{31},\alpha_{32},\alpha_{33}⩾0$$

Since the objective function is in minimization form, then we convert it into maximization form and add the artificial variables as:

(12) $$\mathrm{maximize}\;\rho \left(\chi \right)=-100\alpha_{11}-200\alpha_{12}-300\alpha_{13}-40\alpha_{21}-80\alpha_{22}-120\alpha_{23}-80\alpha_{31}-160\alpha_{32}-40\alpha_{33}$$

(13) $$\begin{array}{c} \alpha_{11}+\alpha_{12}+\alpha_{13}+S_{6}=1,\\ \alpha_{21}+\alpha_{22}+\alpha_{23}+S_{5}=1,\\ \alpha_{31}+\alpha_{32}+\alpha_{33}+S_{4}=1,\\ \alpha_{11}+\alpha_{21}+\alpha_{31}+S_{3}=1,\\ \alpha_{12}+\alpha_{22}+\alpha_{32}+S_{2}=1,\\ \alpha_{13}+\alpha_{23}+\alpha_{33}+S_{1}=1 \end{array}$$

and

(14) $$\alpha_{11},\alpha_{12},\alpha_{13},\alpha_{21},\alpha_{22},\alpha_{23},\alpha_{31},\alpha_{32},\alpha_{33},S_{1},S_{2},S_{3},S_{4},S_{5},S_{6}⩾0$$

In Phase 1 of the two-phase simplex method, we remove the artificial variables and find an initial feasible solution of the original problem which gives the final Tableau in the Table 8.

The basic feasible solution at the end of Phase 1 computation is used as the initial basic feasible solution of the problem. The original objective function is introduced in Phase 2 computation and the usual simplex procedure is used to solve the problem. The Phase 2 gives final optimal value in Table 9.

Similar to the HABBP, the simplex method gives the total optimal cost of assigning cloudlets to virtual machines as 100 s + 120 s + 160 s = 380 s. That is, $cloudlet_{1}$ is assigned to $vm_{1}$, $cloudlet_{3}$ is assigned to $vm_{2}$, and $cloudlet_{2}$ is allocated to $vm_{3}$.

## 5. Virtual Machine Placement Problem

**Figure 2:**
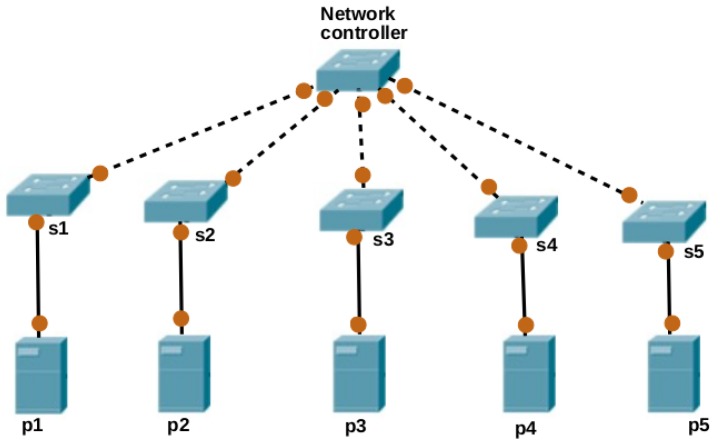
Physical machines and switches in a tree network topology.

In this section, the problem of optimally placing a set of VMs into a set of PMs in the single cloud environment is formulated. As depicted in Figure 2, the tree network topology consists of five PMs and connection points called switches (SWs). The placement of any VM in a PM will be determined by at least a switch node in the figure. In the light of that, there will be huge end-to-end traffic between a given VM and the switch which the VM is dependent on.

It is assumed that the intensity of communication between PMs is negligible compared to the intensity of communication between PMs and SWs. Placing the VMs in PMs that offer an optimal placement cost according to the demands of the VMs will be a major determinant factor in this work. Each PM-SW pair is associated with a cost. Thus, it will not be a good idea to place a VM with intensive demand for a switch in a PM that has a high cost associated with that switch.

The VM placement problem in the data center network can be represented mathematically as a graph G(P,S,E), where *P* is a set of PMs, *S* is a set of SWs, and *E* is a set of links between the PMs and SWs. The links are weighted and represent the cost between any PM-SW pair. In addition, it is also assumed that there is no congestion in the links between the PMs and SWs. The links have enough capacity to handle the switch flow demands of VMs appropriately. More information about the network is as follows.

### 5.1. Parameters

- *P* = $\left\{p_{1},p_{2},\ldots ,p_{n}\right\}$ is a set of PMs.
- *V* = $\left\{v_{1},v_{2},\ldots ,v_{m}\right\}$ is a set of VM requests.
- *S* = $\left\{s_{1},s_{2},\ldots ,s_{k}\right\}$ is a set of switches.
- $l_{s_{h}p_{i}}$ is the latency between $p_{i}$ and $s_{h}$.
- $b_{s_{h}p_{i}}$ is the bandwidth for $p_{i}-s_{h}$ link.
- $\delta_{j}$ represents MIPS of each $v_{j}\in V$.
- $\mu_{i}$ represents MIPS of each $p_{i}\in P$.
- $U_{i}$ represents utilization of $p_{i}$.
- $E_{i}^{idle}$ is the power consumed by $p_{i}$ when it is doing nothing but powered on.
- $E_{i}^{peak}$ is the power consumed when the $p_{i}$ is fully loaded/utilized or at the peak load.

### 5.2. Assumptions

Consider a VM to be placed into PM through the SW in a data center network, the following assumptions are made.

- Each PM has different latency to all SWs in the network.
- Each PM has one and only one link to the SW in the network.
- Each PM can accommodate more than one VM depending on the capacity of the PM.
- Each link between PMs and SWs has enough capacity and there is no congestion on the links.
- The number of VMs, PMs and SWs are equal i.e., n=m=k.

### 5.3. The Mathematical Model

The cost in terms of the time taken to use the $p_{i}-s_{h}$ link is defined as:

(15) $$c_{s_{h}p_{i}}=\frac{vm_{size(j)}}{b_{s_{h}p_{i}}}.$$

where $vm_{size(j)}$ denotes size (MB) of the $v_{j}$ routed through the $p_{i}-s_{h}$ link.

The placement of a $v_{j}$ into a $p_{i}$ depends on the latency between $v_{j}$ and $s_{h}$, and the cost associated with the $p_{i}-s_{h}$ link. Thus, the total cost to place $v_{j}$ into $p_{i}$ through $s_{h}$ is computed as,

(16) $$t_{p_{i}v_{j}}=\beta c_{p_{i}s_{h}}+\alpha l_{s_{h}v_{j}}$$

where β and α∈{0,1} is the weighting for the link and latency. The goal is to place VMs into PMs such that the total placement cost for the PM-SW links consumption and latency between VM and SW is minimized. Thus, an optimization model is defined as follows:

(17) $$\min\sum_{i=1}^{n}\sum_{j=1}^{n}t_{p_{i}v_{j}}x_{v_{j}p_{i}}=\sum_{i=1}^{n}\sum_{j=1}^{n}\left(\beta c_{p_{i}s_{j}}+\alpha l_{s_{i}v_{j}}\right)x_{v_{j}p_{i}}$$

where

(18) $$x_{v_{j}p_{i}}=\left\{\begin{array}{cc} 1, & \mathrm{if}\;v_{j}\;\mathrm{is}\;\mathrm{placed}\;\mathrm{into}\;p_{i},\\ 0, & \mathrm{otherwise}. \end{array}\right.$$

subject to

(19) $$\sum_{i=1}^{n}x_{v_{j}p_{i}}=1,\forall j=1,2,\ldots ,\;n,$$

(20) $$x_{v_{j}p_{i}}\in \left\{0,1\right\},\;\mathrm{for}\;i=1,2,\ldots ,\;n,\;\mathrm{and}\;j=1,2,\ldots ,\;n.$$

(21) $$\sum_{j=1}^{u}\delta_{j}\leq \mu_{i},\;\mathrm{for}\;i=1,2,\ldots ,\;n,\;\mathrm{and}\;u<n.$$

(22) β+α=1

(23) $$c_{s_{j}p_{i}}\geq 0$$

(24) $$l_{s_{i}v_{j}}\geq 0$$

Equation (19) ensures that each VM is mapped to one PM and all VMs are placed. Also, Equation (21) ensures that the total MIPS of VMs placed on a PM should not exceed its capacity. For a given PM, the sum of the MIPS requirements of all VMs placed on it should be less than or equal to the total available capacity of the PM.

Furthermore, it is assumed that there is a linear relationship between the power consumption and utilization of a physical machine in a data center. The energy consumed, $E_{i}$, by a PM $p_{i}\in P$ can be calculated as shown in [28]:

(25) $$E_{i}=E_{i}^{idle}+\left(E_{i}^{peak}-E_{i}^{idle}\right)U_{i}$$

where

(26) $$U_{i}=\frac{\sum_{j\in \gamma_{i}}^{}\delta_{j}}{\mu_{i}}$$

where $\gamma_{i}$ is a set of virtual machines placed on the $p_{i}$.

Thus, the total energy consumed by the PMs after VMs placement can be calculated as

(27) $$\sum_{i=1}^{n}E_{i}=\sum_{i=1}^{n}E_{i}^{idle}+\left(E_{i}^{peak}-E_{i}^{idle}\right)U_{i}$$

## 6. Virtual Machine Placement Algorithmic Solution

The section presents the Genetic Algorithm Based Virtual Machine Placement (GABVMP) for solving the Virtual Machine Placement problem in the cloud computing environment.

### 6.1. Genetic Algorithm Based Virtual Machine Placement

Genetic Algorithm (GA) is a computerized search and optimization algorithm based on the mechanics of natural genetics and natural selection. The GA is proposed by John Holland [29] where each potential solution is encoded in the form of a string and a population of strings is created which is further processed by three operators: Reproduction, crossover, and mutation. Reproduction is a process in which individual strings are copied according to their fitness function. Crossover is the process of swapping the content of two strings at some point(s) with a probability. Lastly, mutation is the process of flipping the value at a particular location in a string with a very low probability.

**Figure 3:**
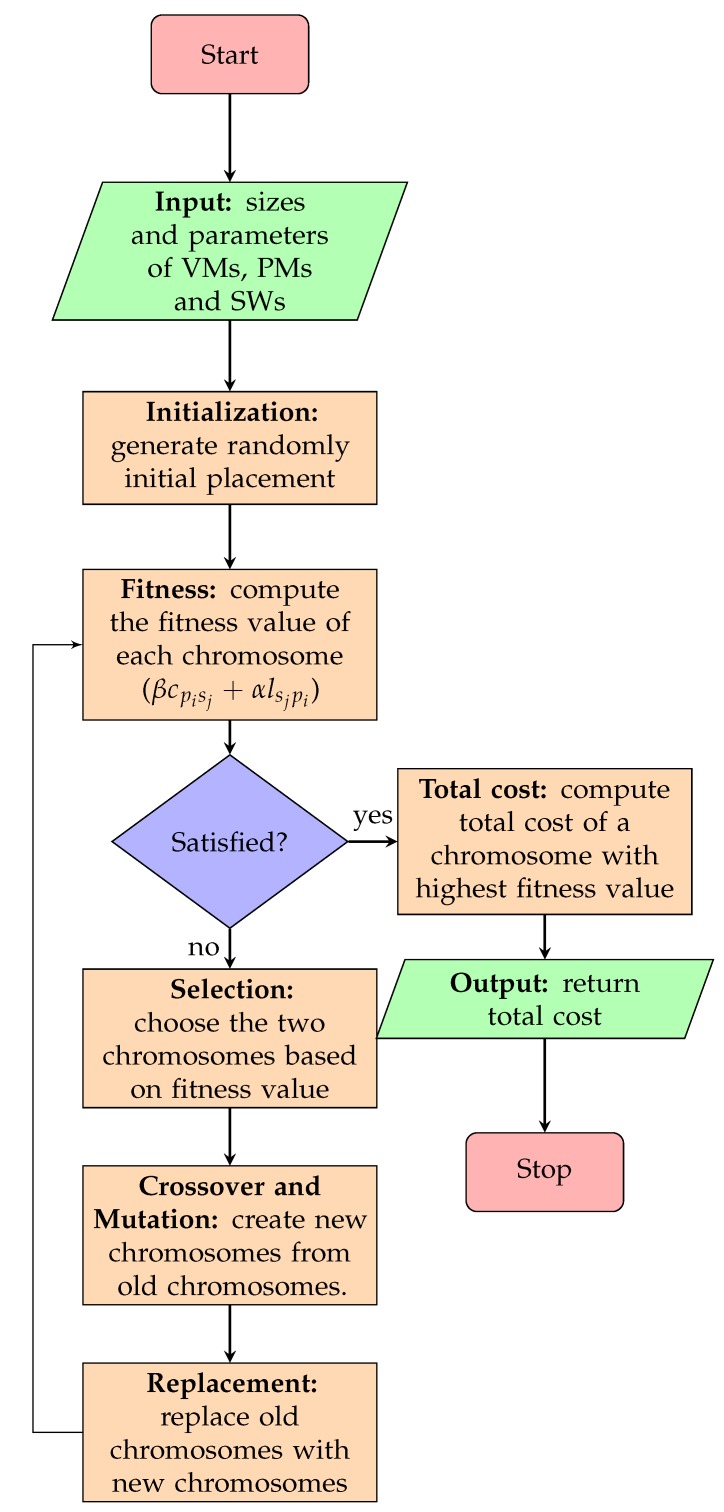
Genetic Algorithm Based Virtual Machine Placement.

Figure 3 describes the GABVMP. The algorithm consists of four parts: Input, initialization, looping and output. In the initialization part, the set of physical machine chromosomes which are also known as population, is generated randomly. The looping part contains fitness evaluation and checks if the optimal solution condition is met according to the optimization objectives. If not, the looping continues, the selection, crossover, mutation and replace functions are applied sequentially. At the end of the loop, the optimal solution will be produced as the output.

### 6.2. Initialization

Each chromosome in the GABVMP contains genes which represent the allocated physical resources and switches to the virtual resources. The value of a gene positive integer representing the identity of the VM being placed in the PM through SW. For Example, let $v_{1},v_{2},v_{3},v_{4},v_{5},v_{6},v_{7},v_{8}$ be a set of VM to be placed in the $p_{1},p_{2},p_{3},p_{4},p_{5},p_{6}$ a set of PM through $s_{1},s_{2},s_{3}$ a set of SW in a data center network. Let’s assume that $p_{1}s_{1},p_{2}s_{1},p_{3}s_{2},p_{4}s_{3},p_{5}s_{3},p_{6}s_{3},p_{7}s_{2},p_{8}s_{1}$ are links between PMs and SWs which is one of the factors to be considered while placing VMs on the PMs. The initial population contains a set of PM-SW link chromosomes where the genes represent the identity of VMs. The initial population is generated randomly by using Algorithm 2.

**Algorithm 2:** Initial population algorithm.

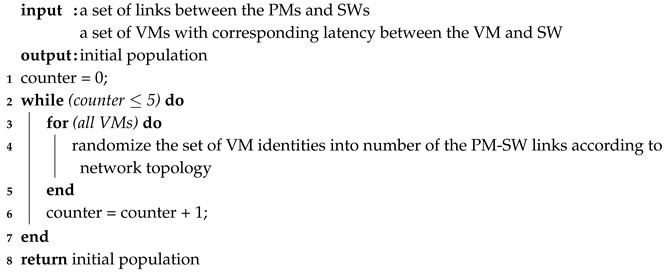



### 6.3. Fitness Evaluation

The objective is to minimise the total cost of placing VMs on the PMs through SWs. As defined in Section 5, The VM placement cost consists of the cost of PM-SW link usage and the latency between the VM and SW. The objective function used by the GABVMP is the same objective function as that of the mathematical model. Thus, the fitness value of each chromosome is calculated as,

(28) $$Fitness\left(chromosome\right)=\beta c_{p_{i}s_{j}}+\alpha l_{s_{j}p_{i}}$$

### 6.4. Generating the Next Population

A new population is generated from an initial population of solutions using their fitness values and genetic operators: Selection, crossover, mutation and reproduction. In order to generate a new population, individuals are selected for participation and the genetic operators are applied as follows.

#### 6.4.1. Selection Process

To select the best chromosomes that would pass their genes into the next generation, the fitness proportionate selection approach is implemented using roulette wheel selection. The fitness function is the total cost of the VM placement represented by each chromosome. The lower the total cost, the fitter the VM placement represented by that chromosome [30]. Thus, the chromosomes with lower values are selected for the generation of the next population.

#### 6.4.2. Crossover Operator

**Figure 4:**
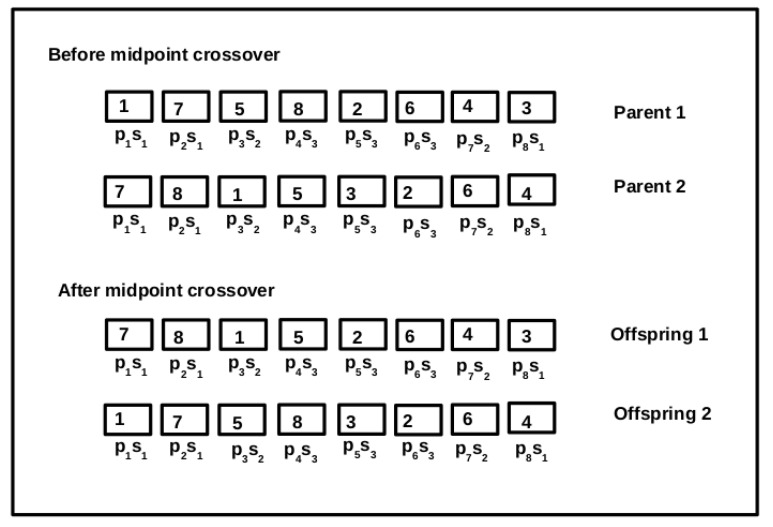
Midpoint crossover.

The crossover operator works on two parent chromosomes and produces a new individual. In GABVMP, a midpoint crossover with crossover probability 0.8 is adopted and crossover operator process is described in Algorithm 3. Figure 4 shows two parent and offspring chromosomes before and after mid crossover respectively.

**Algorithm 3:** Crossover function. **input**: $Q_{1}$, $Q_{2}$: two parent chromosomes **output**: $Q_{\lambda 1}$, $Q_{\lambda 2}$: two offspring chromosomes **1**
Φ = $length(Q_{1})$ **2**
cp = $\frac{Q_{1}}{2}$; **3** mid cross point; **4**
$Q_{\lambda 1}$ = $Q_{1}\left(1:cp\right)UP_{2}\left(cp:\Phi \right)$ **5**
$P_{\lambda 2}$ = $Q_{1}\left(cp:\Phi \right)UQ_{2}\left(1:cp\right)$ **6**
**return**
$Q_{\lambda 1}$, $Q_{\lambda 2}$.

#### 6.4.3. Mutation Operator

**Figure 5:**
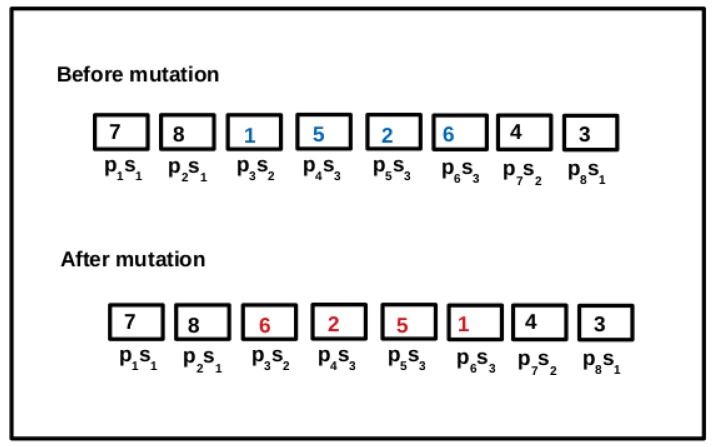
Inversion mutation operation.

In the GABVMP, the next operation is mutation of the offspring. Mutation helps to prevent premature convergence and promotes diversity in the population. In other words, it helps to avoid getting trapped in local solutions. In this work, inversion mutation is adopted where a subset of genes in a chromosome is selected and inverted to form mutated offspring. Figure 5 illustrates the inversion mutation operation on the offspring 1. In offspring 1, a subset of genes (1, 5, 2, 6) in chromosome (7, 8, 1, 5, 2, 6, 4, 3) are selected and inverted to give a new chromosome (7, 8, 6, 2, 5, 1, 4, 3).

#### 6.4.4. Replacement

The replacement operator replaces old chromosomes in the current population with the new chromosomes to form a new population.

#### 6.4.5. Stopping Criterion

GABVMP stops either when the maximum number of generations is reached or the optimal total placement cost is obtained.

The comparison of the GABVMP and the existing related VM placement approaches as mentioned above is presented in Table 10. The comparison parameters include: Latency awareness, energy awareness, network awareness, Internal traffics, flow path allocation and the method adopted to solve the VM placement problem.

## 7. Experimental Results

In this section, we evaluate the performance of our proposed models. We carried out experiments on a desktop computer with specifications: Intel Core i7 CPU @ 2.80 GHz CPU and 4 GB RAM.

### 7.1. Implementation of the Proposed HABBP

**Figure 6:**
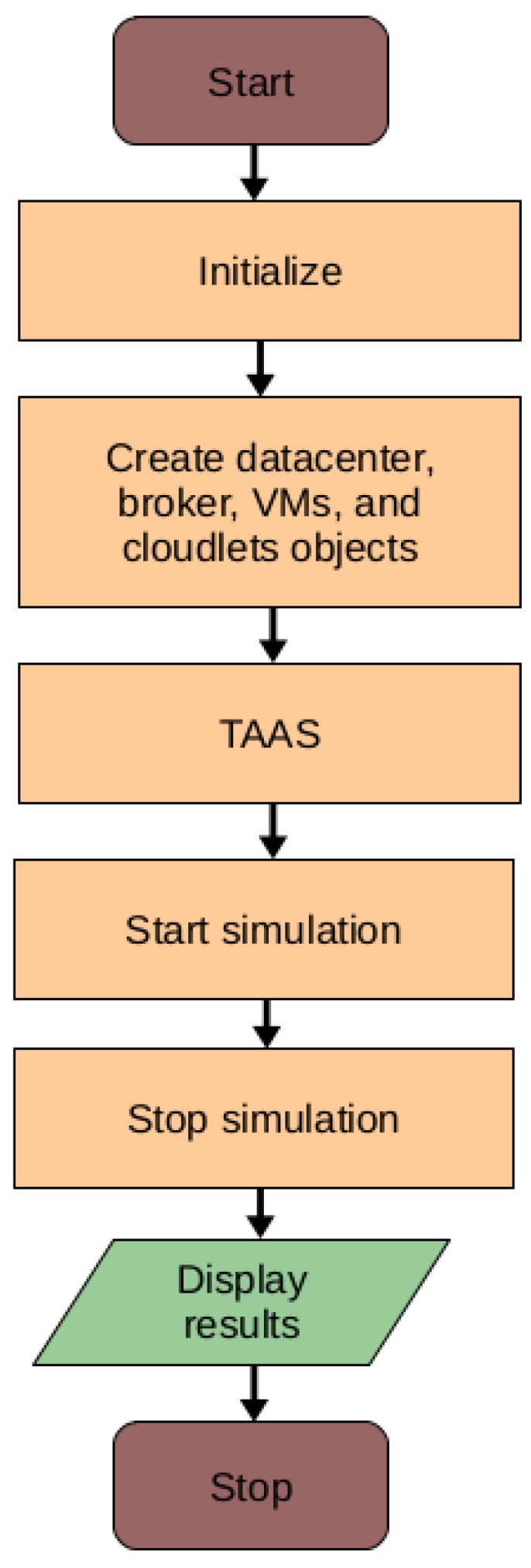
CloudSim Life cycle with HABBP.

We implemented the proposed HABBP on the famous open source cloud simulator known as CloudSim [31] Netbeans IDE 8.2. CloudSim is developed by the Cloud Computing and Distributed Systems (CLOUDS) Laboratory as an extensible Java-based open source framework for modelling and simulation of cloud computing infrastructures and services. Garg et al. [32] refer to CloudSim as an advanced simulator for cloud computing infrastructures and services with great properties such as scaling as well as a low simulation overhead. It provides classes for data centres, VMs, applications, users, computational resources, and scheduling policies. As depicted in Figure 6, there are different stages of the CloudSim life cycle ranging from initialization of cloud infrastructures to the simulation results. Our goal is to validate the performance gains derived from the proposed HABBP compared to the default task assignment strategy as implemented in the CloudSim.

**Figure 7:**
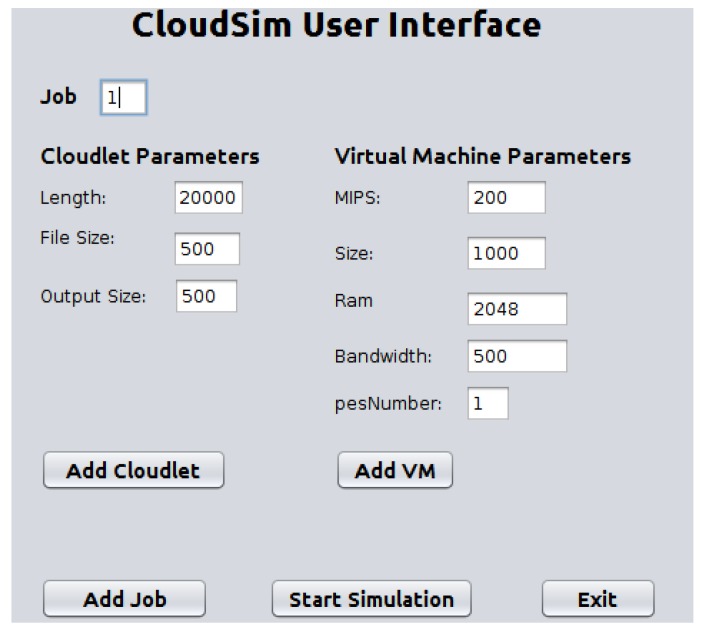
CloudSim User Interface.

The main disadvantage of the current CloudSim, however, is the lack of a graphical user interface (GUI) that allows cloud users to configure cloudlets and the cloud infrastructure parameters, and the lack of optimal cloudlets-to-VMs assignment strategy. In the current work, we extended CloudSim to (i) implement and integrate a graphical user interface using a java *Jframe* class as shown in Figure 7 and (ii) introduced a new cloudlets-to-VMs assignment strategy by creating a new method called *TaskAllocationAlgorithmicSolution()* in the *DatacenterBroker* class of CloudSim.

The model was implemented by assigning each VM to different PMs of the same capacity and all PMs were located in the same data centre. Subsequently, we simulated five jobs using HABBP, default assignment strategy and the Simplex algorithm to assign cloudlets to VMs in CloudSim. Each job had 20 cloudlets and they assigned them to heterogeneous VMs. Each cloudlet and VM had different lengths and MIPS values respectively. We assumed that other parameters, such as file size, output size values of all cloudlets, size, ram, bandwidth and pesNumber of all VMs, were constant, as shown in Table 11 and Table 12.

**Figure 8:**
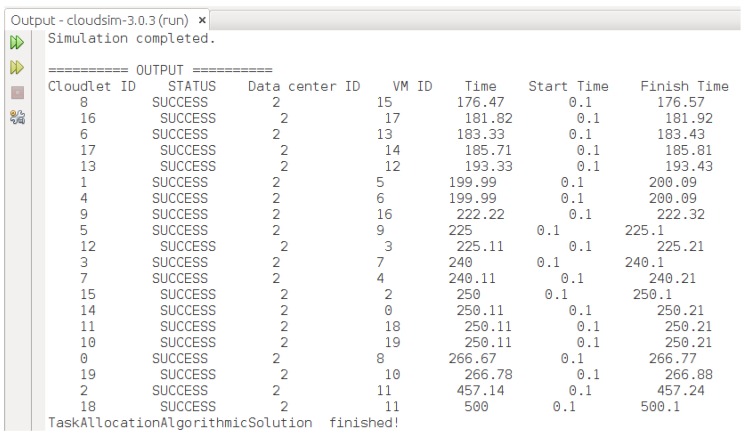
Assigning cloudlets to VMs using HABBP.

**Figure 9:**
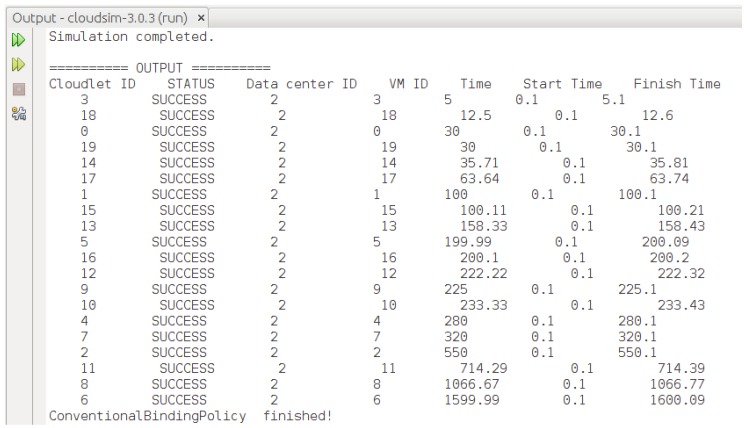
Assigning cloudlets to VMs using default assignment strategy.

**Figure 10:**
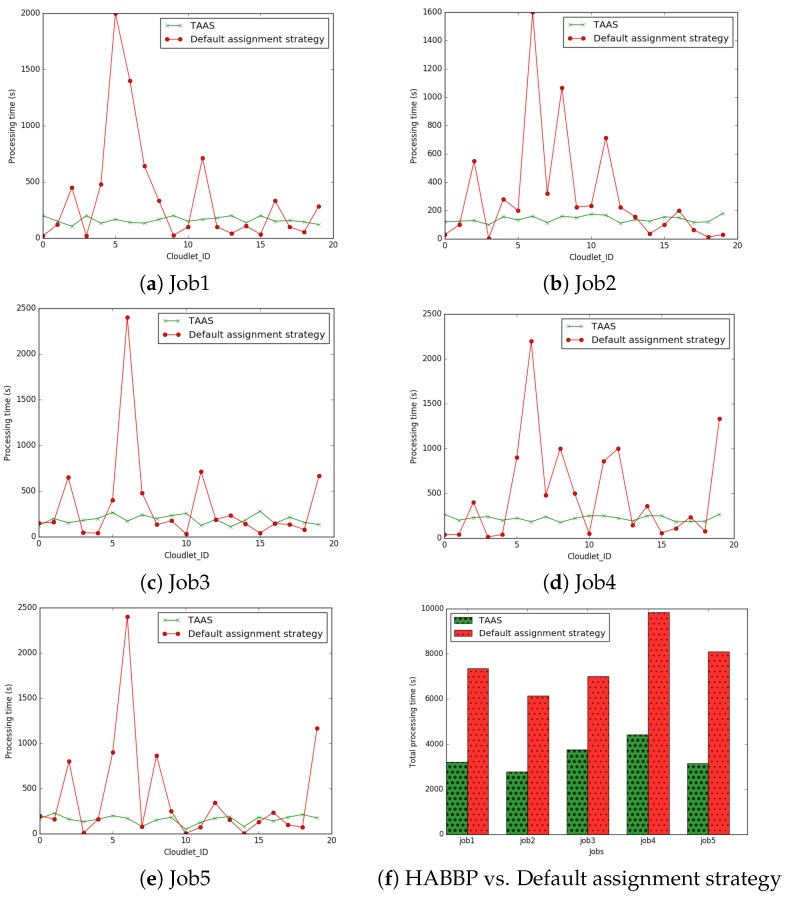
Processing time of the cloudlets in Job 1, Job 2, Job 3, Job 4 and Job 5.

**Figure 11:**
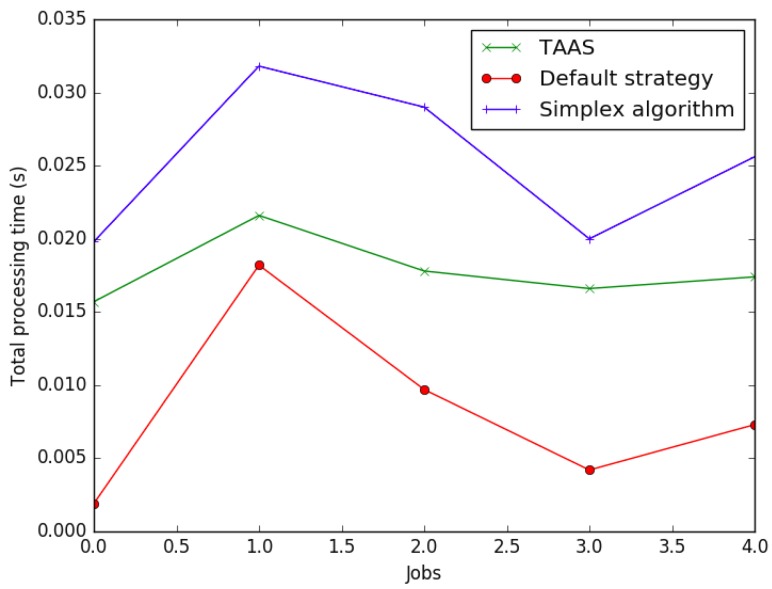
Comparison of computational time.

The simulation results are presented in Table 13 and Table 14, Figure 8 and Figure 9, and plotted in Figure 10 and Figure 11. In job 1, under the default assignment strategy, cloudlets were assigned to the VMs sequentially, that is $cloudlet_{0}$ to $vm_{0}$, $cloudlet_{1}$ to $vm_{1}$, $cloudlet_{2}$ to $vm_{2}$, $cloudlet_{3}$ to $vm_{3}$, $cloudlet_{4}$ to $vm_{4}$, etc. On the other hand, HABBP assigned cloudlets to VMs based on the operations in HABBP. For instance, $cloudlet_{0}$ is assigned to $vm_{8}$, $cloudlet_{1}$ to $vm_{5}$, $cloudlet_{2}$ to $vm_{11}$, $cloudlet_{3}$ to $vm_{7}$, $cloudlet_{4}$ to $vm_{6}$, etc. in job 4; see Figure 8.

In addition, we also compared the overall performance of HABBP with the default assignment strategy and benchmarked both solutions against the Simplex algorithm in terms of the computational time of cloudlets in each job and the total processing time of individual jobs. In Figure 10a–e, it can be seen that some cloudlets took a slightly longer time to complete in HABBP than in the default assignment strategy, while some other cloudlets took a significantly longer time to complete under default assignment strategy than under HABBP. Figure 10f, however, where the total processing time performance of different jobs for HABBP and default assignment strategy is presented, shows that HABBP constantly outperformed the default assignment strategy. Take Job 2 as an example, compared to the default assignment strategy, the total processing time for HABBP was reduced by 54.73% compared with that of the default assignment strategy. HABBP and the Simplex algorithm produced the same optimal allocation cost. HABBP, however, outperformed the Simplex algorithm in terms of computational time as shown in Table 15 and Figure 11.

### 7.2. Implementation of the Proposed GABVMP

In this section, the efficiency of the proposed GABVMP as discussed in Section 6 is evaluated. For the simulation, the mininet module in python3 was used to model the tree topology of the PMs and SWs interconnected in the datacenter. The network topology consisted of equal pairs of PMs and SWs. Each PM-SW link in the network topology had a different capacity in terms of Mbps. The proposed GABVMP and greedy heuristics (Random Placement and First Fit Placement) were implemented and their behaviors were compared on the topology with different numbers of PMs and SWs.

Three experiments were carried out. In the first experiment, 5, 10, 15, 20, 25, 30, 35, 40 VMs were placed on 5, 10, 15, 20, 25, 30, 35, 40 PMs interconnected with the same number of switches using GABVMP with different values of α and β. In the second experiment, 5, 10, 15, 20, 25, 30, 35, 40 VMs were placed on 5, 10, 15, 20, 25, 30, 35, 40 PMs interconnected with the same number of switches using Random Placement with different values of α and β.

In the last experiment, 5, 10, 15, 20, 25, 30, 35, 40 VMs were placed on 5, 10, 15, 20, 25, 30, 35, 40 PMs interconnected with the same number of switches using First Fit Placement with different values of α and β.

**Figure 12:**
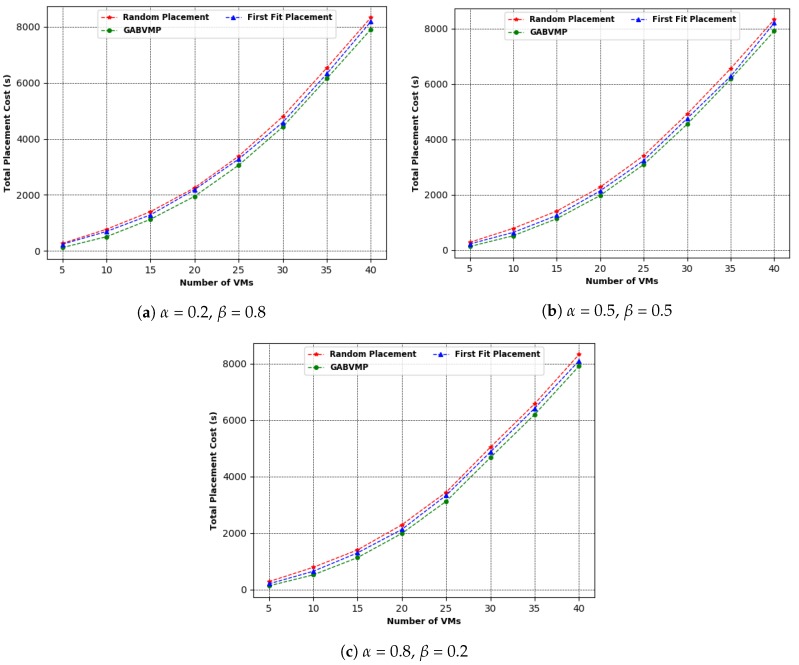
GABVMP vs. Random Placement vs. First Fit Placement.

Figure 12 shows the experimental results of total placement cost in terms of time to implement the proposed GABVMP and the other two existing assignment methods in a data center network with tree topology consisting of 5, 10, 15, 20, 25, 30, 35, 40 of PMs and SWs at different values of of α and β. The GABVMP had a lower cost to place VMs on PMs than the Random Placement and First Fit Placement. For instance, at α = 0.2 and β = 0.8, the Random Placement had a total placement cost of 266 s and the First Fit Placement had a total placement cost of 205 s while GABVMP took a total placement cost of 116 s to place five VMs on five PMs interconnected with five SWs. For α = 0.5 and β = 0.5, the Random Placement had a total placement cost of 275 s and the First Fit Placement had a total placement cost of 235 s while GABVMP took a total cost of 125 s to place five VMs on five PMs interconnected with five SWs. For α = 0.8 and β = 0.2, the Random Placement had a total cost of 284 s and the First Fit Placement had a total placement cost of 216 s while GABVMP took a total cost of 134 s to place five VMs on five PMs interconnected with five SWs.

**Figure 13:**
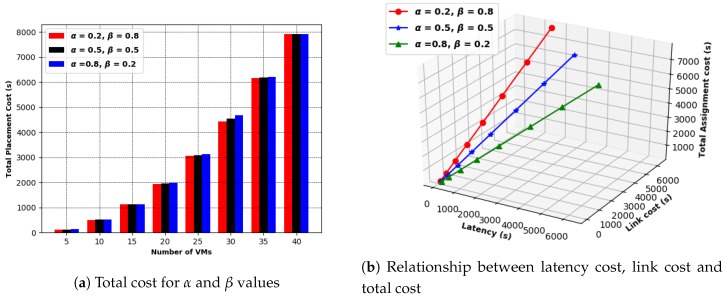
GABVMP for α and β values.

In addition, Figure 13 illustrates the impact of latency on the total assignment cost of the proposed GABVMP. The higher the value of α which denotes the weight of latency, the higher the total placement cost. For instance, when α = 0.2, the total placement cost was 1947 s, when α = 0.5, the total placement cost was 1970 s and α = 0.8, the total placement cost was 1992 s to place 20 VMs into 20 PMs.

**Figure 14:**
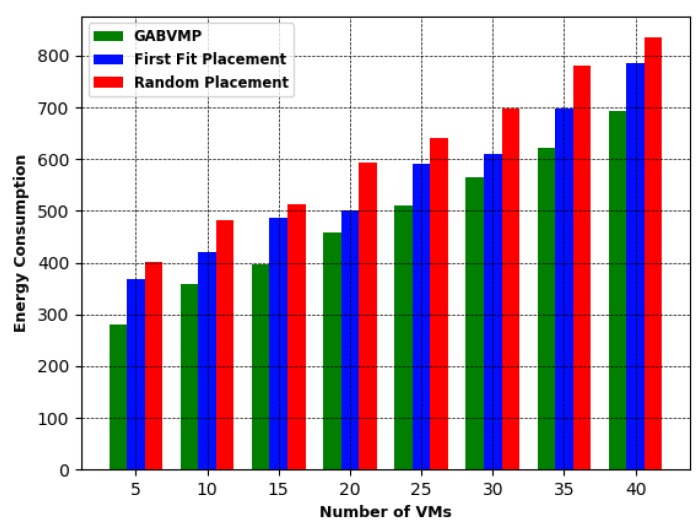
Energy Consumption vs. Number of VMs.

Finally, Figure 14 shows the plot for energy consumption vs. number of VMs. The value of $E_{i}^{peak}$ and $E_{i}^{idle}$ was set to 300 J and 200 J respectively [28]. It is observed from the figure that, when the number of VMs was increased, the energy consumed by the used PMs was also increased. Energy consumption in the proposed GABVMP, however, was lower than the Random Placement method. This is because the number of PMs required to place a given number of VMs was less in GABVMP than the Random Placement and First Fit Placement.

## 8. Conclusions and Future Work

The level of Quality-of-Service in cloud computing is determined to a large extent by the resource allocation strategy adopted. In this work, the issue of Quality-of-Service in cloud computing environments has been revisited. Two solution models have been proposed. Firstly, the tasks-to-virtual machines allocation problem as a linear-programming problem model was formulated and HABBP was proposed, a load balancing policy for binding cloudlets to virtual machines. The simulation results produced by the contributed code to the CloudSim simulation revealed the relative efficiency of the newly proposed HABBP policy in solving and optimizing the virtual resources allocation problem in the cloud computing environment. Secondly, the virtual machine placement problem was presented and proposed a GABVMP as the solution for optimizing the model. The simulation results show that the GABVMP performed better than the two greedy heuristics, Random Placement and First Fit Placement, in terms of PM-SW links consumption which corresponds to the cost of placing VMs on PMs in the data center.

In the near future, the proposed solutions will be used to optimize resource allocation in federated lightweight cloud computing infrastructures targeting not only drought mitigation [33,34] in the rural areas of Africa but also healthcare, following the framework proposed in [35,36]. For such deployments, the policy will be extended to account for traffic engineering characteristics of the cloud computing network for both local traffic [37] and inter-Africa traffic [38] as these can have a large impact on the access to the cloud nodes and thus influence the QoS provided by the cloud. Using UAVs/drones in the context of 5G as proposed in [39] is another alternative for deployment. The implementation of the newly proposed policy in a real and popular cloud computing management platform, such as OpenStack, is another avenue for future work. 

## Figures and Tables

**Table 1:** Cloudlet details.

	${\mathrm{cloudlet}}_{1}$	${\mathrm{cloudlet}}_{2}$	${\mathrm{cloudlet}}_{3}$
id	0	1	2
file-size	500	1000	1000
length	40,000	80,000	120,000
output-size	500	2048	2048

**Table 2:** VM specifications.

	${\mathrm{vm}}_{1}$	${\mathrm{vm}}_{2}$	${\mathrm{vm}}_{3}$
id	0	1	2
size	1000	1000	1000
mips	400	1000	500
ram	2048	2048	2048
pes-number	1	2	2
bandwidth	500	500	500

**Table 3:** Initialize *cost-matrix*.

	${\mathrm{cloudlet}}_{1}$	${\mathrm{cloudlet}}_{2}$	${\mathrm{cloudlet}}_{3}$
$vm_{1}$	100	200	300
$vm_{2}$	40	80	120
$vm_{3}$	80	160	240

**Table 4:** Row *reduced-cost-matrix*.

	${\mathrm{cloudlet}}_{1}$	${\mathrm{cloudlet}}_{2}$	${\mathrm{cloudlet}}_{3}$
$vm_{1}$	0	100	200
$vm_{2}$	0	40	80
$vm_{3}$	0	80	160

**Table 5:** Column *reduced-cost-matrix*.

	${\mathrm{cloudlet}}_{1}$	${\mathrm{cloudlet}}_{2}$	${\mathrm{cloudlet}}_{3}$
$vm_{1}$	0	60	120
$vm_{2}$	0	0	0
$vm_{3}$	0	40	80

**Table 6:** *reduced-cost-matrix*.

	${\mathrm{cloudlet}}_{1}$	${\mathrm{cloudlet}}_{2}$	${\mathrm{cloudlet}}_{3}$
$vm_{1}$	0	20	80
$vm_{2}$	0	0	0
$vm_{3}$	0	0	40

**Table 7:** Optimal Assignment.

Cloudlets	Virtual Machines
${cloudlet}_{1}$	$vm_{1}$
$cloudlet_{2}$	$vm_{3}$
$cloudlet_{3}$	$vm_{2}$

**Table 8:** Phase 1 final Tableau.

Tableau			0	0	0	0	0	0	0	0	0	−1	−1	−1	−1	−1	−1
**Base**	$C_{b}$	$P_{0}$	$P_{1}$	$P_{2}$	$P_{3}$	$P_{4}$	$P_{5}$	$P_{6}$	$P_{7}$	$P_{8}$	$P_{9}$	$P_{10}$	$P_{11}$	$P_{12}$	$P_{13}$	$P_{14}$	$P_{15}$
$P_{2}$	0	0	1	1	0	0	0	−1	0	0	−1	−1	0	0	0	0	1
$P_{3}$	0	1	0	0	1	0	0	1	0	0	1	1	0	0	0	0	0
$P_{13}$	−1	0	0	0	0	0	0	0	0	0	0	−1	−1	−1	1	1	1
$P_{7}$	0	1	0	0	0	0	0	0	1	1	1	1	1	1	0	−1	−1
$P_{4}$	0	0	1	0	0	1	0	0	0	−1	−1	−1	−1	0	0	1	1
$P_{5}$	0	1	−1	0	0	0	1	1	0	1	1	1	1	0	0	0	−1
ρ(χ)		**0**	0	0	0	0	0	0	0	0	0	2	2	2	0	0	0

**Table 9:** Final optimal Tableau.

Tableau			−100	−200	−300	−40	−80	−120	−80	−160	−240
**Base**	$C_{b}$	$P_{0}$	$P_{1}$	$P_{2}$	$P_{3}$	$P_{4}$	$P_{5}$	$P_{6}$	$P_{7}$	$P_{8}$	$P_{9}$
$P_{2}$	−200	0	0	1	1	−1	0	0	−1	0	0
$P_{9}$	−240	0	0	0	1	−1	−1	0	0	0	1
$P_{13}$	−1	0	0	0	0	0	0	0	0	0	0
$P_{8}$	−160	1	0	0	−1	1	1	0	1	1	0
$P_{1}$	−100	1	1	0	0	1	0	0	1	0	0
$P_{6}$	−120	1	0	0	0	1	1	1	0	0	0
ρ(χ)		**−380**	0	0	20	100	40	0	20	0	0

**Table 10:** Comparison of related VM placement problems.

Paper	Latency-Aware	Energy-Aware	Network-Aware	Internal Traffic	Flow Path Allocation	Method Adopted
[18]	No	Yes	No	Yes	No	Scheduling algorithms
[14]	No	No	Yes	Yes	Yes	Greedy method
[15]	No	Yes	No	No	No	EAGLE algorithm
[16]	No	Yes	No	No	No	Ant-colony based algorithm
[20]	No	No	Yes	Yes	Yes	Cluster-and-Cut algorithm
[17]	No	Yes	Yes	Yes	No	Multi-objective evolutionary algorithms
[19]	No	No	Yes	Yes	Yes	Virtual Infrastructure Opportunistic fit (VIO) and VIcinity-BasEd Search (VIBES)
[21]	Yes	No	Yes	Yes	No	Local search algorithm
[22]	No	Yes	No	No	No	Column generation method, cut-and-solve-based algorithm and the call back method
[23]	No	Yes	No	No	No	Neural networks, Self Organizing Map (SOM) and K-Mean Clustering algorithms
GABVMP	Yes	Yes	Yes	Yes	Yes	Genetic algorithm

**Table 11:** A set of jobs.

${\mathrm{cloudlet}}_{\mathrm{id}}$	job1	job2	job3	job4	job5
	(length)	(length)	(length)	(length)	(length)
0	20,000	30,000	150,000	40,000	200,000
1	60,000	50,000	80,000	20,000	80,000
2	90,000	110,000	130,000	80,000	160,000
3	40,000	10,000	90,000	30,000	20,000
4	120,000	70,000	10,000	10,000	40,000
5	200,000	20,000	40,000	90,000	90,000
6	70,000	80,000	120,000	110,000	120,000
7	80,000	40,000	60,000	60,000	10,000
8	50,000	160,000	20,000	150,000	130,000
9	10,000	90,000	70,000	200,000	100,000
10	150,000	350,000	45,000	75,000	5000
11	250,000	250,000	250,000	300,000	25,000
12	45,000	100,000	85,000	450,000	155,000
13	25,000	95,000	140,000	87,000	95,000
14	75,000	25,000	100,000	250,000	4000
15	30,000	85,000	35,000	50,000	110,000
16	300,000	180,000	130,000	100,000	210,000
17	55,000	35,000	75,000	130,000	55,000
18	65,000	15,000	95,000	95,000	85,000
19	85,000	9000	200,000	400,000	350,000

**Table 12:** A set of VMs.

${\mathrm{vm}}_{\mathrm{id}}$	mips value
0	1000
1	500
2	200
3	2000
4	250
5	100
6	50
7	125
8	150
9	400
10	1500
11	350
12	450
13	600
14	700
15	850
16	900
17	550
18	1200
19	300

**Table 13:** Default assignment strategy in CloudSim.

${\mathrm{cloudlet}}_{\mathrm{id}}$	job1		job2		job3		job4		job5	
	${\mathrm{vm}}_{\mathrm{id}}$	proc. time	${\mathrm{vm}}_{\mathrm{id}}$	proc. time	${\mathrm{vm}}_{\mathrm{id}}$	proc. time	${\mathrm{vm}}_{\mathrm{id}}$	proc. time	${\mathrm{vm}}_{\mathrm{id}}$	proc. time
0	0	20	0	30	0	150	0	40	0	200
1	1	120	1	100	1	160	1	40	1	160
2	2	450	2	550	2	650	2	400	2	800
3	3	20	3	5	3	45	3	15	3	10
4	4	480	4	280	4	40	4	40	4	160
5	5	2000	5	200	5	400	5	900	5	900
6	6	1400	6	1600	6	2400	6	2200	6	2400
7	7	640	7	320	7	480	7	480	7	80
8	8	333	8	1066	8	133	8	1000	8	866
9	9	25	9	225	9	175	9	500	9	250
10	10	100	10	233	10	30	10	50	10	3
11	11	714	11	714	11	714	11	857	11	71
12	12	100	12	222	12	189	12	1000	12	344
13	13	42	13	158	13	233	13	145	13	158
14	14	107	14	36	14	143	14	357	14	6
15	15	35	15	100	15	41	15	59	15	129
16	16	333	16	200	16	144	16	111	16	233
17	17	100	17	64	17	136	17	236	17	100
18	18	54	18	13	18	79	18	79	18	71
19	19	283	19	30	19	667	19	1333	19	1167
total processing time	7356		6147		7009		9842		8109	

**Table 14:** HABBP in CloudSim.

${\mathrm{cloudlet}}_{\mathrm{id}}$	job1		job2		job3		job4		job5	
	${\mathrm{vm}}_{\mathrm{id}}$	proc. time	${\mathrm{vm}}_{\mathrm{id}}$	proc. time	${\mathrm{vm}}_{\mathrm{id}}$	proc. time	${\mathrm{vm}}_{\mathrm{id}}$	proc. time	${\mathrm{vm}}_{\mathrm{id}}$	proc. time
0	5	200	4	120	18	125	8	267	18	167
1	9	150	9	125	9	200	5	200	11	229
2	15	106	15	129	15	153	11	229	0	160
3	2	200	5	100	1	180	7	240	8	133
4	16	133	12	156	6	200	6	200	4	160
5	18	167	8	133	8	267	9	225	12	200
6	1	140	1	160	14	171	13	183	14	171
7	13	133	11	114	4	240	4	240	7	80
8	19	167	0	160	5	200	15	176	15	153
9	6	200	13	150	19	233	16	222	17	182
10	0	150	3	175	2	255	19	250	5	50
11	10	167	10	167	3	125	18	250	2	125
12	4	180	16	111	12	189	3	225	16	172
13	7	200	14	136	0	110	12	193	1	190
14	17	136	2	125	17	182	0	250	6	80
15	8	200	17	155	7	280	2	250	13	183
16	3	150	18	150	16	144	17	182	10	140
17	11	157	19	117	11	214	14	186	19	183
18	12	144	7	120	13	158	11	190	9	213
19	14	121	6	180	10	133	10	267	3	175
total processing time	3201		2783		3759		4425		3146	

**Table 15:** Comparison of computational time.

HABBP	Default Assignment Strategy	Simplex Algorithm
0.0157	0.0019	0.0198
0.0216	0.0182	0.0318
0.0178	0.0097	0.0290
0.0166	0.0042	0.0200
0.0174	0.0073	0.0256
